# Toward Digitalization of Maritime Transport?

**DOI:** 10.3390/s19040926

**Published:** 2019-02-22

**Authors:** Pedro-Luis Sanchez-Gonzalez, David Díaz-Gutiérrez, Teresa J. Leo, Luis R. Núñez-Rivas

**Affiliations:** Escuela Técnica Superior de Ingenieros Navales, Universidad Politécnica de Madrid (UPM). Av. Arco de la Victoria, 4, 28040 Madrid, Spain; david.diaz@upm.es (D.D.-G.); teresa.leo.mena@upm.es (T.J.L.); luisramon.nunez@upm.es (L.R.N.-R.)

**Keywords:** additive engineering, artificial intelligence, big data, cloud computing, digitalization, internet of things, maritime transport, robotics, virtual reality

## Abstract

Although maritime transport is the backbone of world commerce, its digitalization lags significantly behind when we consider some basic facts. This work verifies the state-of-the-art as it currently applies to eight digital domains: Autonomous vehicles and robotics; artificial intelligence; big data; virtual reality, augmented and mixed reality; internet of things; the cloud and edge computing; digital security; and 3D printing and additive engineering. It also provides insight into each of the three sectors into which this industry has been divided: Ship design and shipbuilding; shipping; and ports. The work, based on a systematic literature review, demonstrates that there are domains on which almost no formal study has been done thus far and concludes that there are major areas that require attention in terms of research. It also illustrates the increasing interest on the subject, arising from the necessity of raising the maritime transport industry to the same level of digitalization as other industries.

## 1. Introduction

The maritime transport industry is the backbone on which most of global commerce rests. According to the latest data available today from the United Nations Conference on Trade and Development (UNCTAD), it reached 10.7 billion tons in 2017, which is an increase of 411 million tons, of which 50% consisted of dry bulk commodities [1]. Given the current worldwide economic globalization, there must be an agile, safe, and reliable maritime transport chain in order to maintain the levels of economic growth. As the Spanish Association of Shipping Companies, ANAVE, states in its annual 2018 report, the growth of the global seaborne trade was 3.9% in 2017 (expressed in tons), significantly higher than that of 2016, which was 3.0% [2]. Agility, safety, and reliability are some of the topics that must be permanently monitored by any business. These topics can only improve by embracing new technological approaches, one of which is the digitalization of the industry.

Other economic industries are barreling towards digitalization, not just for improving margins and operations, but for pure survival: Financial services, retailers, hinterland transport, etc. Nonetheless, and contrary to what is happening in these other industries, digitalization and Industry X.0 are in their early stages in the maritime transport industry, as the following examples demonstrate:

- An analysis of basic aspects of digitalization, such as the use of interorganizational information systems (IOS), shows that organizations that are involved in Hinterland Transport have a relatively high share of IOS, that is, more than 70%. However, organizations involved in maritime transport have a share of less than 25% [3].
- Although the previous data provides an idea of the steady progress achieved so far at ports and inland infrastructures, ports are lagging behind in regard to the utilization of information technologies/information systems (IT/IS) [4].
- The European Union (EU) lacks a clear strategy towards digitalization in the maritime industry [5].
- Basic security is still more of an aim than a reality, even with the tools of the internet of things (IoT), which are as widely-used as radio frequency identification (RFID), where it is difficult to find commercial systems without critical security flaws and vulnerabilities [6].
- This is also the case with security in the maritime cloud, which is still in its fledging stages [7].

The word digitalization is being used by different people in different ways and in this article, its meaning is that used in the information technology (IT) glossary by Gartner [8]. Digitization and digitalization are two terms that are often confused in the literature and in day-to-day communication; digitization is the process of changing from an analog to a digital format, whereas digitalization is the use of digital technologies to change a business model and provide new revenue and value-producing opportunities, that is, the process of entering a digital business. This work will use digitalization, given its strong relationship with the paradigm of Industry 4.0, which is also addressed in this work.

Any study in this area must specifically emphasize the use of the latest digital technologies, categorized in the following eight domains, which other industries today consider the signs of leading-edge digitalization:

- Autonomous vehicles and robotics (hereafter, robotics); not only in shipyards and ports, but also as aids for sea transport services.
- Artificial intelligence (AI); applications of this technology in combination with big data to make better use of all available information.
- Big data; the productive use of all the information coming from ship sensors.
- Virtual, augmented and mixed reality (VR); tools that are expected to be a part of any vessel equipment.
- The internet of things; today all ships and maritime artifacts are mega-cities, fully-equipped with sensors.
- Cloud and edge computing (hereafter, the cloud); they make the implementation of former domains economically viable.
- Digital security; digitalization demands stronger security tools and processes.
- 3D printing and additive engineering (3DP); they improve maintenance processes.

These eight domains synthesize the technology trends that different analysts like Gartner Group or Forrester are making for the years 2018–2019. The focus is put on the new technology trends on digitalization rather than on a broader IT or technological scope that could include, for example, the use of customer-relationship management (CRM) systems or digital logbooks. These new technology trends are the ones that will make a substantial change in business operation in the coming years.

The maritime transport industry has experienced a drastic increase in operations in recent years, with a growth between 2005 and 2015 of 40% [1], which is especially high considering this period has coincided with an economic downturn. Therefore, the maritime transport industry is one that is considered key for digitalization. Another aspect that contributes to this is the large strides that have been taken towards vessel sensorization in the last ten years. All the equipment, machines, and tools that play a role in the processes of maritime transport (amongst others, cranes, motors, measuring devices from ships) currently provide a large array of data that can be and should be used to optimize processes and operations and, ultimately, improve the economic results of the various participants. The objective of this analysis will be to verify the status of digitalization of those aspects involved in the maritime transport chain and to discover opportunities for improving performance through digitalization.

There are works that have been done on this area so far, but only partially [9]. The contributions that this work provides are:

1. Verification of state-of-the-art regarding the digitalization of the maritime transport industry.
2. Categorization of the status of each of the eight aforementioned domains.
3. Providing an outlook for each of the following three industrial sectors: Ship design and shipbuilding; shipping; and ports. The division of the maritime transport industry into these three sectors has been done based on the need to better assess the industry status, thereby differentiating ship building from ship operations and adding ports as a link to the following step in the logistic chain; that which goes beyond the sea.

A systematic literature review has been used as the main tool for achieving the previous objectives and obtaining a clearer understanding of state-of-the-art regarding digitalization in maritime transport. This work is divided into the following sections: Section 2 describes the methodology used in the study as well as the tools used; Section 3 provides an overview of the state-of-the-art result from applying that methodology; Section 4 entails the analysis and discussion; and finally, Section 5 summarizes the conclusions.

## 2. Approach and Methodology

As mentioned previously, this literature review is systematic, that is, it adopts a replicable, scientific, and transparent process. It uses a detailed technology that aims to minimize bias through exhaustive literature searches of published and unpublished studies and by providing an audited trail of decisions of the reviewer, procedures and conclusions [10].

The search engine INGENIO of the Polytechnic University of Madrid (hereafter, UPM) was used in this work since it provides access to the main databases, such as Cambridge University Press S&T Collection, CERN Document Server, Directory of Open Access Journals (DOAJ) Search Articles, IEEE Xplore, ISSN online, Journal Citation Report, JSTOR, Periodicals Archive Online (ProQuest), ScienceDirect (Elsevier), Scopus, Springer, and the Web of Science.

Rather than generating a single complex Boolean algorithm, the approach applied to this study is of an iterative nature. It used multiple combinations of keywords such as maritime, ship, digitalization, shipping 4.0, maritime 4.0, digitalization, artificial intelligence, virtual reality, mixed reality, augmented reality, robotic, 3D print, big data, cloud computing, edge computing, security, internet of things, internet of ships, autonomous vehicles, unmanned vehicles, additive manufacturing, artificial neural networks, etc. The keywords were determined iteratively by using the results from one search iteration to enhance and improve the search based on the achieved results. For example, starting with 3D print as a key word, it was enhanced to additive engineering and concluded with additive manufacturing. This process was tedious, but best guaranteed the search results. The decision to follow this approach was taken after several trial-and-error attempts, where there was a clear dearth of study on many of the subjects. An approach that reached a single complex Boolean algorithm would increase the risk of overlooking works in some of the areas.

The inclusion of a work in the review of literature was determined by its quality, based on the following parameters:

- Innovation, understood as the discovery of something new based on the scientific work carried out by the authors;
- Use of scientific methods;
- Relevance of the study.

## 3. Digitalization on Maritime Transport: The Situation at the Current Time

The application of the methodology of Section 2 resulted in a total of 2969 published references, which could potentially be related to the area of study. In order to begin the classification of these references, the publication titles were reviewed, thereby discarding those that were clearly unrelated to Maritime Transport digitalization. A second filter was the review of the abstracts, thereby producing a total number of 191 published works, which were subsequently subjected to an in-depth review. This thorough review was performed by reading each of them and determining which ones met the quality criteria described in the approach and methodology section.

To ensure that all relevant works were included, not only were these 191 manuscripts examined, but their references were also reviewed, thereby applying the same approach used with the initial 2969 references. As a result, 23 manuscripts were added to the final list, amounting to a total of 99 published studies.

### 3.1. Autonomous Vehicles and Robotics

#### 3.1.1. Ship Design and Shipbuilding

The two fields with the most studies are the design of unmanned vehicles and robotic applications at shipyards. Most of the work performed in the area of unmanned vehicles (UV) design is related to controlling systems for underwater or surface vehicles.

Commencing with the works on unmanned vehicles involving control systems for underwater vehicles, the 2017 study by Kelasidi et al. [11] proposed a straight-line path following a controller for line-of-sight controlling under irrotational currents of non-predefined directions for underwater snake robots. The study covered both the algorithms and the experimental tests that support them. The work from Zeng et al. [12] presented a non-linear adaptive line-of-sight controller for an underwater UV exposed to ocean currents that addressed the precision improvement of curved path following. Xiang et al. addressed the topic of controlling underwater UV [13], not only on actuated configuration, but also those which are underactuated and use a single framework that can move between two configurations. These last two studies, those by Xiang et al. [13] and by Zeng et al. [12], made use of the Lyapunov theory and backstepping technique.

The work by Narasimhan et al. [14] designed a control system for UV by using the normal force produced by the cambering of fins. Petrich et al. [15] developed equations for an attitude control system that couples pitch and yaw dynamics for autonomous underwater vehicles, thereby testing the results on a small, autonomous UV. To conclude underwater UV controlling systems, the work by Yang et al. [16] generalized the formation controllers designed for particle dynamics to formation controllers for fully actuated UVs with six-degree-of-freedom dynamic models for motions in three-dimensional space.

In regard to controlling systems for unmanned surface vehicles, Ashrafiuon et al. [17] presented the theory and experimental results for controlling sliding, both on surge and lateral tracking. In his 2016 study, Do [18] developed a model for path tracking control for underactuated ships under both deterministic and stochastic loads by using the direct methods of Lyapunov. He also presented the results of the simulation of his method. Kim et al. [19] worked on controlling multiple autonomous ships. Their controlling system for multiple autonomous ships permitted approaching an object without being detected by radar, thereby making use of the line-of-sight control and using MATLAB as software (SW) for simulations. Shin et al. [20] used a dynamic model for controlling unmanned surface vehicles, starting with a three-degree freedom model that was linearized with data arising from experiments to achieve an adaptive control algorithm. Wang et al. [21] developed a mechanism for controlling the approach of a UV to a moving vessel using a 3D path generation based on the real-time, dynamic Dubins-Helix method.

The work done by Naik et al. in 2007 [22] began with the state-dependent Riccati equation technique that provides an effective means of designing non-linear control systems for minimum and non-minimum autonomous phases of UV models. This technique was used to develop a non-linear control model for autonomous UVs. To conclude controlling systems for UV, Shi et al. worked on a literature review on maritime mechatronic systems [23] that included some results in marine control, like developments in terms of control system designs for surface vessels, underwater robotic vehicles, profiling floats, underwater gliders, wave energy converters, and offshore wind turbines.

In our examination of the navigation aids, which is relatively similar to the control systems of UV, the work from Drücker et al. [24] focused on the analysis of impacts between UV gliders and glass-fiber reinforced plastic ships. They used commercial FE-Software Abaqus to develop finite models on the different scenarios, although they disregarded the hydrodynamics between ship and glider for simplification purposes. Smith et al., in their 2011 work [25], provided solutions to the motion planning problem for UV by using the motion equations governing the submerged rigid body applied to the bulbous bow of a ship.

Work has also been performed on algorithms for various purposes. Akçakaya et al. [26] used model-predictive control for establishing point tracking control of variable speed UV. Using classic mechanics, Dong et al. [27] defined a non-linear integral backstepping algorithm that controls the trajectory of an underactuated unmanned marine vehicle. The work developed by Joo et al. in 2015 [28] described a mathematical model that added gliding capabilities to an autonomous UV with certain characteristics, such as quietness and energy saving under low current that distinguishes it from the others. The 2017 study by Mu et al. [29] started from the hydrodynamic maneuvering modeling group (MMG) model of ship maneuvering to establish three degrees of the freedom planar motion model of a podded propulsor for an unmanned surface vehicle.

On auxiliary systems, Ferreira et al. [30] designed and tested a simultaneous localization and mapping (SLAM) framework that provided a relatively rough visual map of the sea floor to support basic navigation and context awareness for an UV. Finally, Sanchez-Lopez et al. [31] presented the design and implementation of a vision-based autonomous landing algorithm using a downward- looking camera for an unmanned aerial vehicle with vertical take-off and landing capabilities.

There are two works on the design of UV which are applicable to environment monitoring. The first is from Vasilijevic et al. [32], which analyzed and designed a cooperative robotic system for environmental monitoring. The second, carried out in 2011 by Lindemuth et al. [33], described the implementation of a combined unmanned surface vehicle that hosts an unmanned aerial vehicle (UAV), thereby proving its adaptability and cost reduction when applied to littoral inspection.

The last study on unmanned vehicles design is that from Rodriguez-Molina et al. [34] that presented a publish/subscribe-based semantic middleware solution that solves the challenge of sending data amongst several heterogeneous autonomous UVs that must communicate underwater in order to complete a global mission in a cooperative manner.

As already mentioned, a relevant number of manuscripts was found which are related to the use of robots in Shipyards and aids for ship inspection. Hover et al. [35] developed several algorithms for central navigation and planning problems on ship hulls to help with in-water ship inspections. They also developed the methodology for using robots in these tasks that make use of the former algorithms. The work from Milella et al. [36] demonstrated the contribution to a better inspection process from ships that use a magnetic autonomous robotic crawler equipped with a low-cost monocular camera. This camera provided images that are especially helpful when used as input to an automatic corrosion detector. Ortiz et al. contributed to vessel inspection [37] with a study that automated the inspection process by using a fleet of robotic agents that supplied images that could teleport the surveyor from a base station to the areas of the hull to be inspected. Their contribution also included an algorithm that, once a set of matches between the features of consecutive frames became available, back-projects features into the ground using previous knowledge of the camera height and the roll and pitch angles, all three of which were supplied by onboard sensors and contribute to robot self-localization.

When discussing contributions to the ship maintenance process, Fernandez-Andres et al. [38] addressed the problems associated with periodical ship blasting with a system based on robots that could meet most of the requirements associated with blasting. Their work also covered a monitoring system and a teleoperations platform that controlled the robots. The work from Iborra et al. [39] presented a family of robots that contributed to hull cleaning with two vertical robotic towers and a robot climber, as well as their control system. Ross et al. designed a robot for stripping paint from ships [40] that accelerated the process, while at the same time being eco-friendly. Finally, in 2017, Xu et al. [41] described an optimized magnetic wheel solution for use in a grit-blasting robot intended to be used on the hulls of ships. A mathematical model was built to derive the exact force requirements considering the mechanical structure of the robot and its position on the hull of the ship, since the magnetic force acting on a magnetic wheel is very sensitive to working conditions.

Paulos et al. developed an aid for the shipbuilding process [42] via a methodology, algorithms, system design, and experiments, thereby addressing self-assembly by large teams of autonomous robotic boats on floating platforms. The originality of their algorithms arose from the possibility of handling multiple docking sites through multiple modules. Sanders et al. also contributed to the shipbuilding process with their 2012 work [43] that designed and tested a robotic system, which combined image processing techniques with a computer aided design (CAD) model. This combination was used to provide information to a multi-intelligent decision module that was involved in the welding process. This system used a rudimentary curvature metric that measured the Euclidean distance between two points in a window with improved accuracy and ease of implementation, especially when identifying images of manufactured parts with distinct corners.

The last robotic manuscript, regarding aids to shipyards, was published by Xu et al. [44], designed a wall-climbing robot for labelling scales for use on oil tankers.

#### 3.1.2. Shipping

In regard to the use of robots in the shipping industry, the manuscript from Zolich et al. [45] reviewed the major advancements on state-of-the-art autonomous maritime vehicles and systems. These were analyzed together with their use in different scenarios, from scientific research to transport, from basic robotic operations to anchoring systems and autonomous vehicles, analyzing the applications for each. It concluded with an analysis of communications options. 

#### 3.1.3. Ports

On the subject of the use of robots in ports, Murphy [46] tested commercial off-the-shelf equipment for ports security in regard to both remotely operated vehicles and autonomous underwater vehicles. The objective was their implementation at Canadian ports in order to improve physical security.

### 3.2. Artificial Intelligence

#### 3.2.1. Ship Design and Shipbuilding

Examining the applications of AI in ship design and shipbuilding, in 2012, Chi-Tsun Cheng et al. [47] proposed a genetic algorithm for designing a path planning for an unmanned underwater vehicle (UnV) that, despite being initially viewed as a suboptimum solution without a full understanding of the problem, had a higher speed and was able to provide solutions at a lower cost. In that same year, Kim et al. [48] applied a Takagi–Sugeno fuzzy model in order to design a stabilization controller system applied to autonomous UnV. The last application based on AI to design an autonomous UnV was that by Zhao et al. from 2014 [49], that designed an adaptive neural network control applied to the control problem of tracking a desired trajectory for a fully actuated marine surface vessel while considering multiple output constraints.

In 2012, Sanders et al. [50] introduced an aid for welding processes in shipyards based on the application of a decision-making system that used multiple parallel artificial intelligence techniques combined with new image capture methods. This system was able to handle the necessary uncertainty whilst still returning a correct weld path.

Kim et al. [51] applied a new neuro-fuzzy technique to estimate the wake field distribution on the propeller plane of a ship. This method proved satisfactory in obtaining safe predictive results. The results from this study may give advanced information to hull form designers for the evaluation and optimization of the stern hull form, by predicting a ship wake distribution at the initial designing stage.

Finally, in this area of AI applications for ship design, Sanders [52] proposed a pattern recognition system for recognizing shipbuilding parts using artificial neural networks and Fourier descriptors. The system used shape contour information that was invariant of size, translation, and rotation. Fourier descriptors provided information and the neural networks made decisions about the shapes.

#### 3.2.2. Shipping

Departing from the studies related to navigational aids in 2015, Hu et al. [53] combined an adaptive fuzzy system with a vector, back-stepping method in order to develop an adaptive fuzzy controller for the dynamic positioning system of vessels. In their work, they used Lyapunov functions to prove that the controller could maintain the vessel at the desired values of its position and heading with arbitrary accuracy, while guaranteeing the ultimate uniform boundedness of all signals in a closed-loop dynamic positioning control system.

Kao et al. developed their study focusing on AI applications in navigational aids [54]. They developed a fuzzy logical method for adding the capability to avoid vessel collisions to the vessel traffic services (VTS) systems for all potential ship collisions in the surveillance area. The proposed alert system for VTS is based on the concept of a danger index (radical axis method) and a fuzzy guarding ring, thereby determining the size of the guarding ring by the fuzzy logical method. The danger index for vessel collision is calculated by the marine geographic information system algorithm.

To help in the identification of ships in an area, a new navigational aid was developed by Rajesh et al. [55] to identify non-linear maneuvering of ships by applying neural network coefficients to determine the non-linear parameters or hydrodynamic derivatives of the maneuvering equations. They used the Levenberg–Marquardt algorithm to obtain network coefficients.

In their 2014 paper, Tsatcha et al. presented their work on this same topic [56], thereby developing a spatial data structure that supported the search for an optimal route between two locations while minimizing cost function. Their work was based on hexagonal meshes and an iterative deepening A* (IDA*) algorithm, thereby providing a bidirectional dynamic routing algorithm and making use of a dynamic graph that facilitates data accessibility. In recent work on AI applied to navigational aids, Zissis et al. [57] made use of artificial neuronal networks to predict ship positioning and to assist with busy port scheduling, vessel route planning, and anomaly detection.

In fleet risk management, there are contributions, such as that from Balmat et al. [58], that proposed an algorithm for the assessment of ship risks based on fuzzy logic, using both static factors (age, flag, gross tonnage, number of companies, duration of detention, and type), as well as dynamic factors (sea state, windspeed, visibility). The work from Sii et al. [59] is another application of fuzzy logic to this same subject. Their work focused on the development and representation of linguistic variables to model risk levels subjectively. These variables were then quantified by using fuzzy set theory. Finally, the last study on fleet risk management, performed by Liu et al. [60], used case-based reasoning for the design of an SW that provided assistance with collision avoidance by using historical data from the records of maritime affairs.

Lyridis et al. [61] developed improved financial fleet management by using artificial neural networks based on the feed forward back error propagation algorithm to predict the freight market of tankers. In 2012, Wen et al. [62] developed a system that used artificial neural networks and logistic regression to conduct classification and prediction of fishing vessels involved in smuggling.

#### 3.2.3. Ports

Similar to robotics, ports is the least developed sector in terms of studies. Fancello et al. [63] contributed an aid to ports management with this work in which they proposed two algorithms: A dynamic learning predictive algorithm based on neural networks and an optimization algorithm for resource allocation. They sought to use them to reduce the uncertainty interval regarding the arrival of ships in port as well as optimizing the entire workday, while considering actual demand and the operations of the terminal.

In 2013, Cubillos et al. worked [64] on a pattern recognition system for recognizing shipbuilding parts using artificial neural networks and Fourier descriptors. The system used shape contour information that had an invariable size, translation, and rotation. Fourier descriptors provided information and the neural networks made decisions about the shapes. Finally, Lalla-Ruiz et al. [65] used AI to generate an algorithm for berth location.

### 3.3. Big Data

#### 3.3.1. Ship Design and Shipbuilding

No studies were found in this industrial sector within this domain.

#### 3.3.2. Shipping

Regarding navigation and big data, in 2017 Isenor et al. [66] used open-sourced technologies for the design and implementation of a maritime data management infrastructure capable of coping with vessel positional data and supporting vessel metadata. In 2018, Kang et al. [67] made use of big data techniques to manage automatic identification systems (AIS) and extract conclusions on maritime transport management in the Singapore Strait. They concluded that ship speed generally decreases with the increase of ship density; the four classic traffic flow models for the fifteen legs in the Singapore Strait are estimated by the weighted and non-weighted least square approaches, respectively. They were able to estimate the theoretical ship traffic capacity for each segment in the Singapore Strait.

Furthermore, applying big data techniques to navigation data from the Singapore Strait, Chen et al. [68] designed principal fairways in this crowded strait based on ship trajectory observations of transit-passage and cross-strait transits.

The manuscript from Lei [69] also dealt with the same matter. It closely explored an automated method for mining trajectory data and detecting anomalies. It considered the indications of anomalous behavior that happens at unusual location points and sub-trajectories in the spatial domain as well as in the sequence and the way in which these locations and sub-trajectories occur. The proposed model described movement behavior and detected outlying features in trajectory data. It also developed an anomaly detection algorithm based on this model in which an indicator was used to evaluate suspicious behavior and rate trajectory behavior according to the defined outlying features.

Making advances with studies on big data applications to navigation, Li et al. [70] developed an algorithm that made use of big data to optimize escape routes in order to avoid collisions between ships. This study used big data analysis to evaluate the degree of risk of collision between ships and calculate the minimum and maximum intervals of last-minute action in dangerous collision situations. To evaluate the degree of risk, the degree of ship collisions, and the danger level, they also calculated the ranges of the initial time of last-minute actions. Three evaluation functions of ship sailing were calculated by using this degree of risk of ship collision and the intelligent avoidance of a shipwreck was performed by genetic algorithm.

Park et al. [71] proposed a backend system capable of storing and searching for various kinds and large amounts of S-100-based data to support e-navigation services. The S-100 is a hydrographic geospatial standard that supports integration and efficient utilization of various marine data. The International Maritime Organization (IMO) introduced the concept of the common maritime data structure (CMDS) and adopted the IHO S-100, a universal hydrographic data model, as a baseline of the CMDS.

Finally, in regard to navigation works that make use of big data, Santamaria et al. [72] developed a system that uses the large amount of data generated by Sentinel-1 radar to track unidentified ships.

In regard to maritime economics, Anan et al. [73] combined big data and AI to visualize ship performance on the real sea with a high degree of accuracy and, through the application of the results to weather routing simulation, could greatly improve the fuel economy.

The 2016 work from de Souza et al. [74] proposed the use of data mining techniques over satellite AIS data to generate an algorithm inspired by studies on animal movement for analyzing the location where fishing ships are operating. Using this algorithm can prioritize and enforce measures on fisheries management and conservation worldwide.

Kim et al. [75] proposed a data-driven method for the early detection of vessel delay. Using a large amount of vessel tracking data from real-time satellite-automatic identification systems (S-AIS), combined with historical shipping data, they built a framework of refined case-based reasoning. The proposed method also provided a process for analyzing the causes of delays by matching the tracking patterns of real-time shipments with those of historical shipping data.

The last contribution to maritime economics arises from Perera et al. [76], who proposed an intelligence-based machine for handling the large amount of data on ship performance and navigation to improve the quality of their respective navigation strategies. The framework was divided into two main sections of pre- and post-processing. The data pre-processing was an onboard application that consisted of sensor faults detection, data classification, and data compression steps. The post-processing was a shore-based application (i.e., in data centers) and consisted of data expansion, integrity verification, and data regression steps.

Yang et al. [77] developed a cooperative cognitive maritime big data system for enhancing communications amongst vessels.

#### 3.3.3. Ports

Fernandez et al. [78] designed an application to improve ports management. The SW enables a meaningful visualization and helps to make decisions using techniques like data acquisition, transfer, storage, big data analysis, and information visualization applied to meteorological stations, maritime buoys, and AIS data from vessels. It used the architecture from the European Union Future Internet platform (FIWARE). Subsequently in 2018, this team [79] used FIRWARE to build a geographic information systems (GIS) application.

Heilig et al. [80,81] provided an overview on the state-of-the-art of digital transformation in seaports, together with the historical evolution that ports have experienced in their digitalization. In these studies, they illustrated the use in ports like the Hamburg one of big data technics, combined with IoT and other technics.

### 3.4. Virtual, Augmented, and Mixed Reality

#### 3.4.1. Ship Design and Shipbuilding

To assisting with training shipyard workers, Lee et al. [82] designed and implemented a system for the task of spray-painting. As an immersive display, they used a projection-based wall-type display and a head-mounted display (HMD). To provide stereo images to the user, they proposed circular polarizing filters. To match the visualization parameters of a virtual scene to a physical space, a point-matching-based calibration method was developed for an optical see-through HMD configuration.

In 2018, Blanco-Novoa et al. [83] evaluated existing commercial augmented reality (AR) applications in order to apply them to shipbuilding. They detailed those which were considered the most relevant, as well as the main use cases for the application at shipyards. Subsequently, they described the system of Navantia, a Spanish shipyard, in order to determine its performance in a shipyard workshop and inside a ship under construction. Two AR software development kits were selected: Vuforia and ARToolkit. They concluded that the ARToolKit reading distances were much longer than those obtained by Vuforia under normal lighting conditions. In addition, it was concluded that the ARToolKit, since it is open-source, was a better long-term choice than other proprietary solutions when developing applications for industrial AR (IAR) devices, because of the possibility of extending the software.

Bukhari et al. [84] presented an algorithm for VR generation that was not complex to use. They applied it to the creation of a multipurpose fuzzy semantic enhanced 3D virtual reality simulator, which was used for the design of underwater robots. The architecture of the simulator was based on fuzzy domain ontology and natural language processing (NLP).

The study from Kostas et al. [85] presented a system based on VR for ship design focused on people and ship safety. It is of interest because of the enhancements introduced for managing the movements of people and ships during emergency scenarios.

In 2014, Mesing et al. [86] produced a development architecture for VR systems and applied them to the naval industry. Their architecture built a virtual environment, using a variety of sources (XML, texts, videos, etc.) as input, which is especially important in the naval industry given the need of scene enrichment with dynamic aspects. They can include these aspects and sources using an authoring approach.

Von Lukas et al. [87] included different proofs of concepts of augmented eeality applied to the naval industry. This work included use-cases on sales and marketing, product validation, design and production, and harbor surveillance. Finally, Vasilijević et al. [88] performed a review of the existing augmented reality applications with a marine application.

#### 3.4.2. Shipping

Cooke et al. [89] described a proof of concept interactive 3D simulator developed to narrow the training gap between classroom teaching and a bridge simulator assessment of virtual reality. Its differentiation and key functionality in terms of visualization, physics/interaction, and game mechanics are influenced by consideration of pedagogical learning models during requirement capture. The focus was on collision avoidance.

In 2015, Grabowski [90] proposed applications of wearable, immersive augmented reality into ship navigation, a technology that is linked to the local area, a broad area, and virtual private networks, as well as to shipboard and shoreside sensors, which can provide local and transit-specific knowledge for track-keeping, maneuvering, and collision avoidance. It could also receive real-time weather, visibility, and vessel speed restriction information for a specific transit in advance of the transit or in real-time and link that information to the existing decision-support bridge systems.

In conclusion, we mention the work from Hugues et al. [91], who developed an algorithm for using augmented reality to detect and track navigation horizons in combination in a single display with the video stream of electronic charts.

#### 3.4.3. Ports

No studies were found in this industrial sector within this domain.

### 3.5. The Internet of Things

#### 3.5.1. Ship Design and Shipbuilding

Fraga-Lamas et al. [92] compared different identification systems (quick response (QR) codes, RFID, etc.) to shipyard requirements, such as water resistance and corrosion response. The final aim was to design and implement a system for identifying, tracking, tracing, and controlling pipes in a shipyard.

The work of Ang et al. [93] focused on the fact that Industry 4.0 applied to ship design and shipbuilding can generate more efficient vessels in terms of energy consumption. To do this, they first drew parallels between ship design, manufacturing and operation processes, then identified key challenges, proposed a closed-loop ship life cycle framework, and finally, outlined potential future directions in smart design, manufacturing, and operation of ships in an industry 4.0 value chain in order to achieve more energy-efficient vessels.

#### 3.5.2. Shipping

Choi et al. in 2018 [94] examined shipping economics. More specifically, their work presented a framework for an IoT-based container tracking system that, making use of the standard mobile network, enabled users to monitor the international flow of container movement as well as to achieve smooth cross-border procedures. It also discussed the policy development and international cooperation that should take place to enable the introduction of this container tracking system.

Katayama et al. [95] also contributed to shipping management by testing the different commercial RFID systems available for maritime purposes, thereby discussing their pros and cons. This work covered both active and passive RFIDs. They finally presented their developments on new types of active RFID tags with low power consumption, providing long-life battery operation. Similarly, in 2017, Tian et al. [96] undertook an analysis of existing IoT applications with the aim of introducing them into this industrial sector.

Finally, the manuscript from Park et al. [97] provided aids for navigation based on IoT through the design and implementation of a mobile middleware that facilitated the exchange of ship information between vessel traffic services.

#### 3.5.3. Ports

Goudarzi et al. [98] designed a system based on the IoT using RFIDs with the objective of better managing port warehouses. The main issues addressed were scalability, fault tolerance, and privacy. The architecture was simulated by commercial SW packages OMNet++ and OverSim. Similarly, Shi et al. [99] also contributed to ports management with their study on the use of the IoT, based on RFIDs applied to a container terminal.

The last manuscript applying the IoT to ports is that from Heilig et al. [4], who analyzed IoT applications for ports within the context of a global analysis made on information systems used at seaports.

### 3.6. Cloud and Edge Computing

#### 3.6.1. Ship Design and Shipbuilding

Lee et al. [100] designed a cloud-based system for the ship inspection process based on data capture from RFIDs. Their work explained the applicability of the proposed method to existing inspection/maintenance systems, thereby offering a systematic solution and providing a working technology demonstration of the approach.

#### 3.6.2. Shipping

Dellios et al. [101] defined an implementation plan for a maritime cloud as well as the delivery model for such architecture. Yang et al. [102] proposed an approach to cloud on a software defined network (SDN) that improved performance and monitoring order to handle scheduling issues. The focus was on analyzing the efficiency of a communication network based on SDN and Fog-based communications from a mathematical perspective.

#### 3.6.3. Ports

The 2017 study from Heilig et al. [103] proposed two greedy heuristic and two hybrid-simulated annealing algorithms for route optimization between terminals. This algorithm was used to build a cloud-based system. The Heilig et al. [104] work in 2014 specified a cloud-based SOA that assisted with port operations. Their focus was on inter-terminal operations.

### 3.7. Digital Security

#### 3.7.1. Ship Design and Shipbuilding

No studies were found in this industrial sector within this domain.

#### 3.7.2. Shipping

Fernandez-Carames et al. [6] developed a methodology for security validation on RFID. This work analyzed the main security issues, examined the different RFID systems, and detailed the main type of cyber-attacks. In 2017, Lee et al. [7] proposed the synchronization method from Almanac as a safety method when applied to a maritime cloud for synchronizing different vessels. Finally, Xing et al. [105] developed an algorithm for improving safety on board.

#### 3.7.3. Ports

No studies were found in this industrial sector within this domain.

### 3.8. 3D Printing and Additive Manufacturing

#### 3.8.1. Ship Design and Shipbuilding

No studies were found in this industrial sector within this domain.

#### 3.8.2. Shipping

The work from Chen [106] analyzed 3DP from an economical perspective. The modeling predicted that the total demand of international transport would decline after the application of 3D printing. In consumer countries, the return of manufacturing would increase its container business. In resource countries, resource exports would decline while container business would grow due to the local processing of printing filaments.

#### 3.8.3. Ports

No studies were found in this industrial sector within this domain.

## 4. Analysis and Discussion

Although study on digitalization is not new, there are topics that are being intensively researched, whereas others are clearly under-studied. As can be seen in Table 1, robotics is the domain most widely studied, whereas AI, big data, virtual reality and IoT have a relevant number of works. Cloud, digital security and 3DP are the least researched domains.

Table 2 presents these studies per digital domain and per the industrial sectors mentioned in Section 1. The table is broken down into eight sections in order to better illustrate the situation in each domain. The analysis and discussion will focus on each of the eight domains and three sectors.

Analyzing this data in detail and focusing first on robotics (Table 2a), we find that 34 of the 36 works have been performed on ship design and shipbuilding. These 34 works include 24 dedicated to the design of unmanned vehicles, whereas the remaining ten works are devoted to the use of robots at shipyards.

Out of the 24 on unmanned vehicles design, 13 are dedicated to control systems and 11 are related to systems or algorithms for controlling these UV, both for underwater vehicles [11,12,13,14,15,16] as well as for surface ones [17,18,19,20,21]. The last two works on UV control systems are on the design of a non-linear control system [22] and a literature review on maritime mechatronics systems [23].

Another group of works, focusing on the study of unmanned vehicles design, are relatively related to UV control systems, navigation aids, that include two works [24,25].

The remaining nine cover a variety of topics:

- Four are algorithms for various purposes including [26] speed control, [27] trajectory control, [28] providing gliding capabilities, and [29] design of a propulsor;
- Two address the subject of the design of auxiliary systems [30,31];
- Two manuscripts revolve around the design of UV with applications for environment monitoring [32,33];
- The last specifies a middleware for UV [34].

As already mentioned, there are ten works related to the use of robots at shipyards. Three of the ten are developing aids to ship inspection [35,36,37]. Four are related to the ship maintenance process [38,39,40,41]. Two develop an aid to the shipbuilding process [42,43] and the last concerns the use of robots for a very specific task regarding oil tankers [44].

To conclude, in regard to robotics, only one work has been found on the use of robots in ports [46] and one on their use in shipping [45]. This low number of studies indicates a gap for new studies.

In regard to AI (Table 2b), a higher granularity on topics is found. However, it is interesting to note that in this digitalization domain, most of the works are related to the shipping industry; Ten of the 19 works pertain to AI in the maritime industry, whereas on robotics, there was only one. Of these ten, five are studying matters related to navigational aids [53,54,55,56,57]; three are works on risk management [58,59,60]; one is an AI application for maritime economics [61]; and the last is on using AI to improve security in shipping [62].

As with robotics, ports is the sector least developed by AI. Only three of the remaining nine are related to port management [63,64,65]. When examining the application of AI to ship design and shipbuilding, three are algorithms for the construction of unmanned vehicles [47,48,49], one is an application from AI aiming to aid shipyards [50], and the last two are AI developments applicable to ship design systems [51,52].

When we examine the works found on big data (Table 2c), the situation is similar to that of AI. Most of the studies focus on shipping, with none on ship design and shipbuilding. There are four studies that address ports. The result for this last industrial sector is rather predictable since there is not an abundance of applications upfront that can be considered the use of big data for ship design and shipbuilding processes, although some may be developed in order to be able to improve these processes. The absence of big data studies on ports is not so obvious, since the use of existing data could improve process execution.

The four works mentioned on ports are [78,79,80,81], that deal with applications to port management.

The 12 works on big data applied to shipping can be summarized as follows:

- Seven are applications on navigation [66,67,68,69,70,71,72].
- Four are works on maritime economics [73,74,75,76].
- One is a very specific study on the use of big data for enhancing communications amongst vessels [77].

Thus far, the domains covered are the more widely studied. Robotics, AI and big data comprise 70% of the works found. The remaining five topics, virtual reality, IoT, cloud, digital security and 3DP, are seldom or practically never studied. Virtual reality and IoT are the topics of ten and nine works, respectively. Virtual reality (Table 2d) is not studied in any paper on ports. Seven papers concern ship design and shipbuilding, that is, applications to shipyards [82,83]; applications to the SW for design purposes [84,85,86]; and generic studies on the application of virtual reality to the ship design and shipbuilding process [87,88]. There are three dedicated to shipping navigational aids [89,90,91].

The nine works on IoT (Table 2e) are distributed quite homogenously among the three industrial sectors. There are four studies dedicated to shipping, of which [94,95] are developments for assisting management; [97] provides navigation aids based on IoT; and [96] is an analysis of existing applications, which attempt to introduce them to this industrial sector. Of those on IoT applied to ports, two focus on improvements to port management as a main goal [98,99], whereas one includes analysis of IoT applications for ports [4].

The remaining two on IoT cover ship design and shipbuilding, which mainly facilitate the operations of shipyards [92,93].

Cloud (Table 2f) is a domain that provides many opportunities for study, due to the absence of works found and the relevant interest of institutions such as the EU. Two works were found on applications to port management [103,104]. An additional one [100] is a cloud application to ship design and shipbuilding (more specifically, to the inspection process). The last two are works on shipping; a work to develop an implementation plan for a maritime cloud [101] and an another on fog-based communications [102].

The last two domains are those on digital security and 3DP, which have been studied in three works and one work, respectively. Digital security (Table 2g) is under-studied, although this situation should change given its relevance at the present time and the technological possibilities arising from blockchain and quantum computing. Only three papers were found on shipping security design [6,7,105].

Finally, 3DP (Table 2h) is the domain which is practically not studied at all with only one work on maritime economics [106].

The results on the eight domains prove the lack of studies in some of them, evident in digital security and in 3DP. This conclusion opens streams for future research in these areas that should be extended to other domains and industrial sectors. This is also the case for ports, AI, VR, and big data.

**Figure 1:**
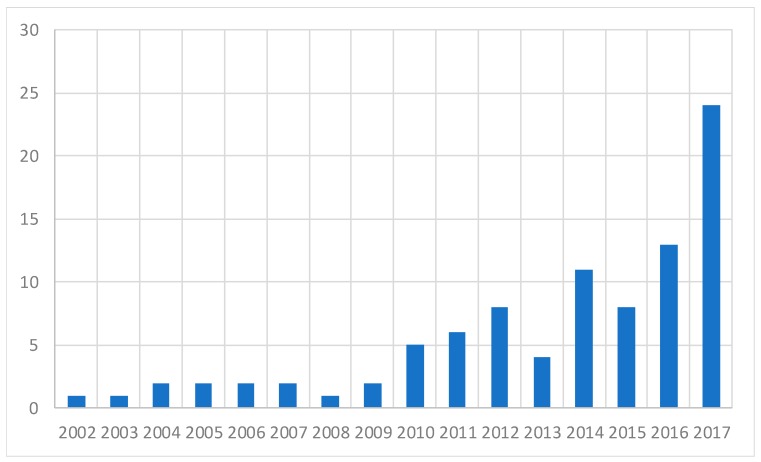
Publication trend on maritime digitalization published per year.

The evolution of studies per year of publication can be found in Figure 1. This indicates an increasing interest on the subject, which results from the need to raise the maritime transport industry to the level of digitalization of other industries.

**Figure 2:**
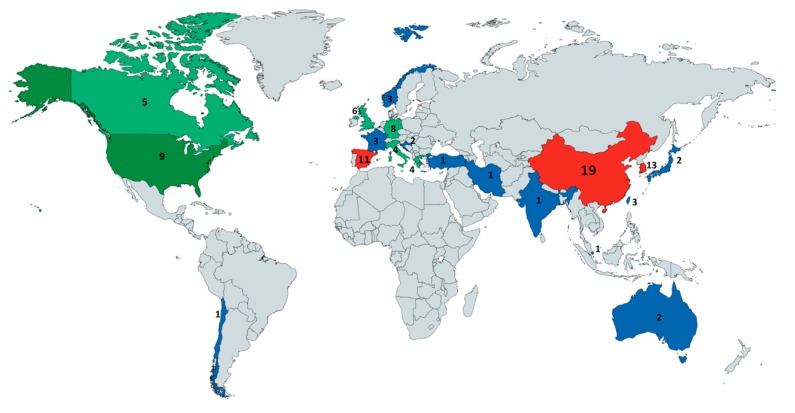
The number of studies on maritime digitalization produced by country (generated using https://mapchart.net/). Countries in red are those with the most papers. Those in green have produced an intermediate amount and in blue are those with the fewest number of studies.

The geographical distribution of these works can be seen in Figure 2. China, Korea, and Spain are the leading countries in terms of work on maritime transport digitalization studies. It also indicates that Europe is clearly paving the path on digitalization study, with almost 50% of the works originating from this continent. China, Korea, and Europe have the largest maritime companies worldwide, which explains the interest in studying how to improve their operations with technological waves, such as digitalization.

## 5. Conclusions

This work makes use of a literature review as method for stating the present situation on digitalization of maritime transport, as well as for uncovering research streams that need to be worked. Accordingly, a systematic literature review has been performed on the major journal databases (amongst others and as mentioned in Section 2, JCR, IEEE Xplore, and ProQuest). More than 2900 works were examined in order to compile a final list of 99, which met the quality criteria mentioned in Section 2.

The main conclusions are:

- The most widely-studied domains are robotics, artificial intelligence and big data, especially unmanned vehicles in robotics and the use of artificial intelligence as a means of supporting vessels aids for navigation.
- Cloud, security, and 3DP have room for study, given their low number of works found.
- There are sectors within the most widely studied domains that have still not been explored, such as the use of robotics in sea transport services, the integration of the studies done on AI in the industry, and the use of big data on ship design and shipbuilding processes.
- When examining the different industrial sectors, ports is the sector which offers great opportunities for studies given that it needs to interact with inland transport, which is highly digitalized. In regard to shipping, while domains such as AI and big data have been extensively studied, domains such as robotics and IoT, which can have a significant impact on shipping operations, are underdeveloped and merit closer attention. When considering ship design and shipbuilding, the situation is similar, but in terms of the different domains, robotics has been extensively studied, whereas the remaining domains are practically ignored or not studied at all.

Answering the question that titles this work, when looking to the published studies, maritime transport is moving towards digitalization at different speeds in the different domains and industrial sectors defined in Section 1. While robotics has been investigated in detail, mostly on UV design, there are domains practically unexplored. This opens new streams that deserve work and attention. This work and attention should revert to the industry like it has happened with the studies done so far on UV design. Those studies have made possible the development of the ferry ‘Folgefonn’, owned by Norwegian operator Norled. This ferry has been recently tested in the presence of the Norwegian Maritime Authority for navigation using its automated dock-to-dock solution.

Therefore, this work proposes several domains that need further dedication and which look promising in terms of their applications to the maritime industry. This work has found a good number of applications, going from pure navigation aids to algorithms that can prioritize and enforce measures on fisheries management and conservation worldwide. There can be others that could contribute to many areas of concern in the maritime industry, for example, maritime disaster prevention. Additive manufacturing can reduce costs significantly both for shipyards as well as for pure sea transport services by shipping companies. Cloud can also play a significant role for these companies in regard to the many aspects of their operations, such as global access to data from a fleet and unified procedures for freight contracting and tracking. Finally, digital security must progress significantly for the safe implementation of the other digital domains.

## Figures and Tables

**Table 1:** References per domain.

Domain	Number of Manuscripts
Robotics	36
Artificial Intelligence	19
Big Data	16
Virtual Reality	10
IoT	9
Cloud	5
Security	3
3DP	1
TOTAL	99

**Table 2:** Number of studies per digital domain and maritime transport sector.

Maritime Transport Sector	Number of Manuscripts							
	(a) Robotics	(b) AI	(c) Big Data	(d) Virtual Reality	(e) IoT	(f) Cloud	(g) Security	(h) 3DP
Design and Shipbuilding	34	6	0	7	2	1	0	0
Shipping	1	10	12	3	4	2	3	1
Ports	1	3	4	0	3	2	0	0
TOTAL	36	19	16	10	9	5	3	1

## Abbreviations

3DP	3D Printing and Additive Engineering
AI	Artificial Intelligence
AIS	Automatic Identification Systems
ANAVE	Spanish Association of Shipping Companies (from the Spanish “Asociación de Navieros Españoles”)
AR	Augmented Reality
CAD	Computer Aided Design
CMDS	Common Maritime Data Structure
Cloud	Cloud and Edge Computing
CRM	Customer Relationship Management
EU	European Union
FIWARE	European Union Future Internet platform
GIS	Geographic Information Systems
HMD	Head-Mounted Display
IAR	Industrial Augmented Reality
IDA*	Iterative Deepening A* algorithm
IMO	International Maritime Organization
IOS	Inter-organizational information systems
IoT	Internet of Things
IT	Information Technology
IT/IS	Information Technologies/Information Systems
MMG	Maneuvering Modeling Group
NLP	natural language processing
QR	Quick Response code
RFID	Radio Frequency Identification
Robotics	Autonomous Vehicles and Robotics
S-AIS	Satellite-Automatic Identification Systems
SDN	Software Defined Network
SLAM	Simultaneous Localization and Mapping
SOA	Service Oriented Architecture
SW	Software
UNCTAD	United Nations Conference on Trade and Development
UV	Unmanned vehicles
UAV	Unmanned aerial vehicle
UPM	Polytechnic University of Madrid (from the Spanish “Universidad Politécnica de Madrid”)
UnV	Underwater vehicles
VR	Virtual Reality, Augmented Reality and Mixed Reality
VTS	Vessel Traffic Services
